# Biogenic synthesis and characterization of antimicrobial, antioxidant, and antihemolytic zinc oxide nanoparticles from *Desertifilum* sp. TN-15 cell extract

**DOI:** 10.1186/s11671-024-04076-8

**Published:** 2024-10-02

**Authors:** Taswar Nadeem, Muhammad Kaleem, Lubna Anjum Minhas, Saima Batool, Muhammad Muzamil Sattar, Rifat Bashir, Abdul Samad Mumtaz

**Affiliations:** Department of Plant Sciences, Faculty of Biological Sciences, Quaid-i-Azam University, Islamabad, 45320 Pakistan

**Keywords:** *Desertifilum* sp. TN-15, Microscopic analysis, Polyphasic approach, Antimicrobial, Biomedical applications, ZnO–NPs

## Abstract

Cyanobacteria, being a prominent category of phototrophic organism, exhibit substantial potential as a valuable source of bioactive compounds and phytonutrients, including liposomes, amino derivatives, proteins, and carotenoids. In this investigation, a polyphasic approach was employed to isolate and characterize a newly discovered cyanobacterial strain from a rice field in the Garh Moor district of Jhang. *Desertifilum* sp. TN-15, a unique and less explored cyanobacterial strain, holds significant promise as a novel candidate for the synthesis of nanoparticles. This noticeable research gap underscores the novelty and untapped potential of *Desertifilum* sp. TN-15 in the field of nanomedicine. The characterization of the biogenically synthesized ZnO–NPs involved the application of diverse analytical techniques. Ultraviolet–visible spectroscopy revealed a surface plasmon resonance peak at 298 nm. Fourier transform infrared spectral analysis was utilized to confirm the involvement of biomolecules in the biogenic synthesis and stability. Scanning electron microscopy was employed to probe the surface morphology of the biogenic ZnO–NPs unveiling their size of 94.80 nm and star-shaped. Furthermore, X-ray diffraction analysis substantiated the crystalline nature of ZnO–NPs, with a crystalline size measuring 46 nm. To assess the physical stability of ZnO–NPs, zeta potential and dynamic light scattering measurements were conducted, yielding values of + 31.6 mV, and 94.80 nm, respectively, indicative of favorable stability. The antibacterial capabilities of *Desertifilum* sp. TN-15 are attributed to its abundance of bioactive components, including proteins, liposomes, amino derivatives, and carotenoids. Through the synthesis of zinc oxide nanoparticles (ZnO–NPs) with this strain, we have effectively used these chemicals to generate nanoparticles that exhibit noteworthy antibacterial activity against Staphylococcus aureus (MIC: 30.05 ± 0.003 µg/ml). Additionally, the ZnO–NPs displayed potent antifungal activity and antioxidant properties, as well as significant antihemolytic effects on red blood cells (IC_50_: 4.8 µg/ml). Cytotoxicity assessment using brine shrimps revealed an IC_50_ value of 3.1 µg/ml. The multifaceted actions of the biogenically synthesized ZnO–NPs underscore their potential applications in pharmacological and therapeutic fields. This study proposes a novel method for ZnO–NPs production utilizing the recently identified cyanobacterial strain *Desertifilum* sp. TN-15, highlighting the growing significance of biological systems in the environmentally friendly fabrication of metallic oxide nanomaterials.

## Introduction

Nanotechnology (NT) is regarded to be at the frontline of the invention of nanomaterials, which are employed in numerous domains of science and technology [1]. NT entails creating useful nanoparticles (NPs) for the biomedical field, particularly for drug delivery [2]. Fe is used widely in both biological and geological processes, making it one of the most important infrastructures in use today [3]. Nanoscience has captured the interest of scientific communities across the world over the preceding ten years due to its prospective applications in the pharmaceutical, medical diagnostics, and disease-treating industries, as well as in the energy, electronics, agricultural, chemical, and aerospace industries [4–6]. By 2030, it is anticipated that the worldwide nanomaterials market will expand by 20%. The creation of natural biological product access points for the synthesis of environmentally responsible tiny particles has been proposed in light of encouraging results from physicochemical-based nanoparticle synthesis [7–12]. Metal NPs have already been successfully synthesized by utilizing various chemicals. Noteworthy examples include silver (Ag) [13–15], gold (Au) [16], zinc (Zn) [17–19], copper (Cu), titanium (Ti) [20–25], cadmium (Cd) [26], iron (Fe) [27–30] and alginate [31]. Zinc oxide NPs can be produced using a variety of chemical, biological, and physical techniques. Zinc oxide nanoparticles have been produced chemically using wet chemical, electro-deposition, chemical micro-emulsion, spray pyrolysis, [31, 32], microwave-assisted combustion, chemical precipitation, and direct precipitation, according to the literature. Physical techniques involve the use of strong vacuums in procedures as molecular bean epitaxy, thermal evaporation, and pulsed laser deposition [33]. Of them, biological processes are economical and environmentally beneficial for producing zinc oxide nanoparticles [34]. Zinc oxide nanoparticles (NPs) are produced by "green synthesis," which involves the use of plant extract, fungi, bacteria, and microorganisms [35].

Cyanobacteria are the most interesting microorganisms for the production of nanoparticles compared to all other microorganisms because they can produce unique chemicals that have significant biotechnological applications [36, 37]. Systems for producing biomass and cyanobacterial culture grow and reach their maximum biomass capacity much faster than crop plants [38]. Cyanobacteria have been used to create Pd, Ag, Au, and Pt nanoparticles either extracellularly or intracellularly [32, 39]. ZnO–NPs have also been synthesized using EA03 and *Arthrospira platensis* [36, 40]. A wide range of secondary metabolites, such as vitamins, phycobiliproteins, antioxidant enzymes, and phenolic compounds, are abundant in cyanobacteria and are frequently employed in antibacterial, antifungal, Bioremediation [33–35], anticancer[36], and anti-HIV medications [41]. Since cyanobacteria cell extract is a rich source of chemicals including amino, hydroxyl, and carboxyl groups, it can be utilized as a metal oxide coating and reducing agent for the creation of nanoparticles [42].

Current research on the antimicrobial properties of nanomaterials indicates that they reduce negative effects, albeit outcomes are restricted and controversial. Nanoparticles' antimicrobial effectiveness is due to their microscopic shape, improved electronics/optional characteristics, and greater surface area to volume proportion, which results in an improved field of chemistry and dynamic curtailment effects [37]. Due to their low-profit margins, several pharmaceutical companies have lost interest in creating novel antibiotic molecules. Thus, it is crucial to create effective and affordable techniques to produce curative agents to combat the aforementioned health difficulties [38]. Impressive advancements in nanoscience and nanotechnology, particularly in diagnosis and therapy, are encouraging for addressing the issue of the spread of antibacterial medication resistance and improving life quality. Physico-chemically designed biocompatible nanomaterials can offer effective treatments for infectious and cancerous diseases [38–40]. It is important to consider the fact that pathogenic bacteria strains cannot develop a resistance to micron-sized particles when assessing the antimicrobial efficacy of generated nanomaterials [43, 44].

In this work, we sought to investigate the novel potential of cyanobacterial extract from *Desertifilum* sp. TN-15 as a stabilizing and reductive agent for the manufacture of zinc oxide nanoparticles (ZnO–NPs). We concentrated on identifying the special qualities of these nanoparticles and assessing their antibacterial activity against clinically significant microorganisms, such as the fungus *Alternaria alternata* and the bacteria *Escherichia coli*, *Pseudomonas aeruginosa*, *Bacillus subtilis*, and *Staphylococcus aureus*. Furthermore, we looked at ZnO–NPs' cytotoxic potential and antioxidant ability in relation to human red blood cells and Artemia salina eggs. With the help of *Desertifilum* sp. TN-15 extract, ZnO–NPs that have been synthesized with new goals in mind are being investigated for potential biomedical uses.

## Materials and methods

### Sample collection site

**Fig. 1:**
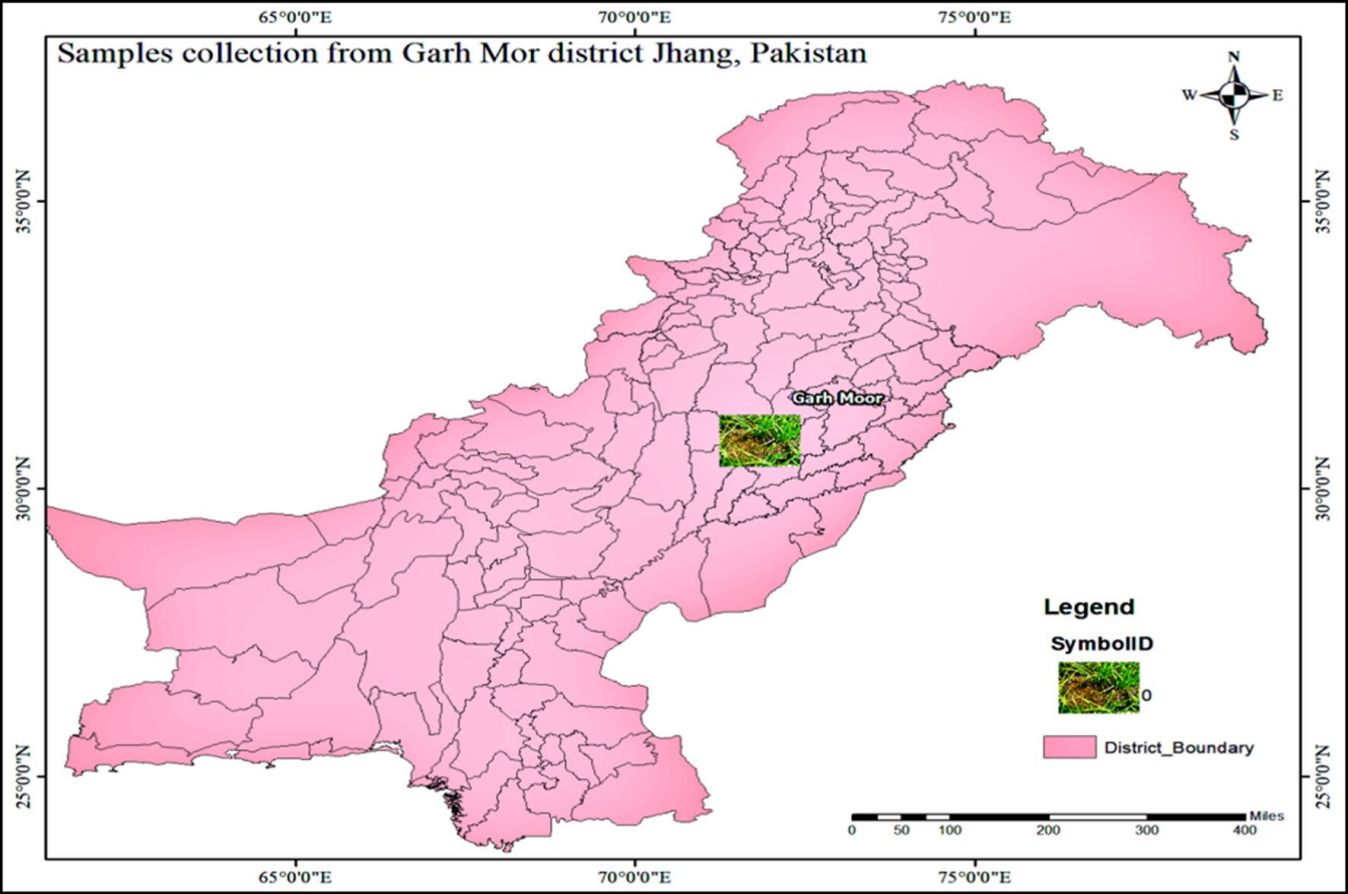
Map of collections sites of algal samples

In May 2022, samples were gathered from the rice fields in Jhang district, Punjab, Pakistan, to isolate certain strains. Water from tubewells and canals served as the sample's resources. The obtained sample's temperature (41 °C), EC (350), pH (7.11), longitude (71.86), and latitude (30.82) are displayed in Fig. 1.

### Isolation, purification, and growth of cyanobacterial strain *Desertifilum* sp. TN-15 through standard culture methods

The algae samples were inoculated on solid BG_11_ agar media. Then incubated at 27 °C for seven to ten days with constantly illuminated by white lights under a comparatively medium illumination range (400–500 lux). Growth of the cyanobacteria sample in 3–5 days appeared on solid BG_11_ media, and their dark green, pale green or blue-green filamentous colony formed and observed in the light microscope. Various techniques are employed to produce cyanobacterial purify culture, depending on the cyanobacterial morphology (filament-like), cell shape and dimensions, and level of mobility. Because cyanobacteria may have such interactions with other microbes, a pure culture is not always feasible. Axenic filamentous cyanobacterial cultures were made using the single filament method. In the single filament method, Isolations were performed using a pipette to repeatedly move individual cells or threads of cyanobacteria on a liquid or solid substrate in Petri plates.

For purification, the isolated cyanobacterial strain was put in the BG-_11_ agar and allowed to move freely. Next, use a light microscope to examine any microbiological contaminants linked to the isolated filamentous cyanobacteria. The isolated cyanobacteria were then transferred to a flask containing 250 ml of liquid BG-_11_ medium (pH 7.3). For proper growth of isolated strains, every freshly isolated cyanobacteria flask was kept in a growing chamber at 27 °C under fluorescent white lighting.

### Morphological analysis of cyanobacterial strain *Desertifilum* sp. TN-15

Cyanobacteria strains were analyzed morphologically using a light microscope and imaged with a 64 MP, PDAF camera. The characteristics studied included cell length and width, cell filament sheath shape, and mobility present. The most recent taxonomic literature was used to identify the strains using the conventional method [35, 41, 42, 45, 46].

### Molecular identification of cyanobacterial strain *Desertifilum* sp. TN-15

The isolated strain of cyanobacteria was identified by 16S rRNA sequencing [47]. For 25–30 days, the isolated strain was cultured in BG-_11_ liquid. After centrifuging the liquid culture for 10 min at 6000 rpm, the biomass was collected, rinsed again with 0.85% NaCl saline solution, and genomic DNA was obtained using the CTAB technique. Agarose gel electrophoresis was used to confirm the isolated DNA's purity. The Cyanobacteria-specific primers CYA 781 R(a)e and CYA 359 F were used to amplify the 16S rRNA gene fragments. Thermal cycler PCR was used for the amplification stage. The 16S rRNA gene was exposed to the following PCR conditions: 12 min of initial denaturation at 95 °C, 30 cycles of 94 °C for 1 min, 55 °C for 1 min, and 72 °C for 2 min, and one extension step at 72 °C for 10 min. The PCR products were validated using agarose gel electrophoresis before being purified using a gel extraction kit. The sequences were analyzed with BLAST V2.0 software (http://www.ncbi.nlm.nih.gov/BLAST).

### Biomass collection of cyanobacterial strain *Desertifilum* sp. TN-15

During the late exponential phase (days 15–21), the culture-purified Cyanobacterial strain was obtained by centrifugation at 6000 rpm for 10–15 min. The resultant cells were then rinsed three times with distilled water before all medium debris was removed. The cell extracts were made using the collected biomass.

### Preparation of cyanobacterial strain *Desertifilum* sp. TN-15 cell extract

The organic matter was gathered and dried at 45 °C for 12 h in the oven to get a consistent weight. Next, a sterile mortar and pestle were used to smash the dry biomass until it was uniformly smooth. Before usage, the biomass powder was kept in falcon vials that were well sealed at 4 °C. The dried 1 g biomass was dissolved in 300 ml of solvent and then incubated at 60 °C for 24 h. After 24 h, the fluid cell extract was passed through a Whatman No.1 filter paper. Throughout succeeding processes, the cell extract was maintained at 4 °C.

### Biosynthesis of zinc oxide nanoparticles (ZnO–NPs) from cyanobacterial strain *Desertifilum* sp. TN-15

For the manufacture of ZnO–NPs, the filtrate 50 ml cell extract was mixed drop by drop into 50 ml of a 0.02 M Aqueous Zinc acetate dihydrate solution (Zn(CH_3_CO_2_)2H_2_O) using a magnetic stirrer at the constant speed [17]. After 10 min of stirring, 0.1 M NaOH (pH 7–9) was added to the constantly churning solution, drop by drop, until a white solution formed. The resulting white solution was vigorously stirred for 2 h before being centrifuged at 5000 rpm for 15 min. The resultant pellet was washed with 15 ml deionized water at least 2 times to remove the free debris from the resultant pellets. The pellet was then dried for 14 h at 60° C in an oven, collecting the dried fine powder, which was then preserved for further analysis in new Eppendorf tubes or an airtight microtube.

### Physical characterization of synthesized ZnO–NPs

#### Visual color change test

The visible color shifts from green to white throughout the incubating period allowed researchers to identify the production of zinc oxide nanoparticles.

#### Ultraviolet–visible spectroscopy (UV) analysis of NPs

A double-beam UV–visible spectrophotometer (UV-4000 spectrophotometer, Berlin, Germany) was configured with a 1 nm resolution to acquire the UV–visible spectra of the biosynthesized nanoparticles. The test solution was then placed in a quartz cuvette, and its optical density was measured at wavelengths ranging from 200 to 600 nm. The graph was generated by plotting wavelength (X-axis) against absorbance (Y-axis) [43, 48].

#### Fourier transform infrared spectroscopy (FTIR) analysis of NPs

In order to create a pellet and analyze the dried powder form of synthesized zinc oxide nanoparticles from aqueous fruit extract, potassium bromide (Kbr) was added. This allowed for the possible presence of bioactive components on the surface of the zinc oxide nanoparticles. Using an FT-IR (Thermo scientific Fisher Nicolete IS10, Waltham, MA, USA) instrument, the spectra data were acquired in the 400–4000 cm^−1^ resolution range in order to identify the functional groups that were present in the sample [27, 49, 50].

#### X-ray diffraction (XRD) analysis of NPs

In order to determine the grain size and crystallinity of the biosynthesized ZnO–NPs, the dried powder form was subjected to X-ray diffraction (XRD) analysis. This involved placing the ZnO–NPs in a sample holder and then using an X-ray diffractometer (PANalytical, Almelo, The Netherlands, Europe) to record the spectral patterns. The XRD machine operated at 40 kV and used a current of 30 mA with Cu Kα radiation, with an angle of 2θ ranging from 30° to 90° [51–53].

#### Scanning electron microscopy (SEM), and energy dispersive X-ray spectroscopy (EDX) analysis of NPs

The topology and elemental compositions of ZnO–NPs were ascertained using (SM5910 JEOL, Tokyo, Japan) scanning electron microscopy (SEM) in conjunction with an energy dispersive X-ray (EDS) apparatus. In short, ZnO–NPs were applied to the stub using carbon tape, fixed, and then covered in gold by sputtering. After that, the loaded stub was put inside the instrument chamber to be analyzed [44].

#### Zeta potential analysis of ZnO–NPs

Zeta potential analysis was used in a colloidal system to determine the surface charge of ZnO–NPs. After ultrasonication, a homogenous suspension of nanoparticles was created, and it was centrifuged for 20 min at 6000 rpm. After that, it was examined at 3.4 eV using a nano analyzer (Malvern Zetasizer Nano, Tokyo, Japan). DLS measurement was used to examine the dispersing type and average size of ZnO–NPs [54, 55].

### Evaluation of biosynthesized ZnO–NPs for biomedical applications

#### Antibacterial efficiency of ZnO–NPs

The Agar well diffusion method was used to assess the antibacterial activity of the ZnO–NPs. All of the pathogenic strains, including *Bacillus subtilis, Pseudomonas aeruginosa, Escherichia coli*, and *Staphylococcus aureus*, were acquired from the Quaid-i-Azam University's biological department in Islamabad, Pakistan. The procedure of dilution broth was utilized to determine minimal inhibitory concentrations (MICs) [14]. To create uniform lawns, bacterial strains were grown on standard nutritional agar media (100 μl of calibrated suspension with an optical density of 0.5). On the agar plates, wells with various ZnO–NPs concentrations (50, 100, 150, 200 µg/ml) and sterile borer (6 mm) sizes were created. Additionally, Ampicillin standard borer (5 mg) was utilized. The zones of inhibition (ZOI) were evaluated after a 24-h incubation period at 37 °C.

$$\text{Percentage Inhibition}=\left[\frac{{\textrm{Control}}-{\textrm{Treatment}}}{{\textrm{Control }}}\right] \times 100$$

The symbols C and T represent specific parameters related to the growth inhibition assessment.

#### Antifungal efficiency of ZnO–NPs

The antifungal efficiency of synthesized ZnO–NPs was tested against *Alternaria alternata* [32]. On PDA media, a preserved culture of fungus was cultivated for seven days at a temperature of 26 ± 1 °C. The antifungal activity of synthesized zinc oxide nanoparticles was evaluated using the poisoned food approach. The manufactured ZnO–NPs concentrations (50 to 200 μg/ml) were added to PDA media for this purpose. A Cork borer was used to insert *Alternaria alternata* (6 mm) inoculum disc into the center of PDA plates that had been modified with nanoparticles. PDA Agar Media without nanoparticles served as a form of positive control. The inoculated petri plates were cultured for 7 days at 26 ± 1 °C, and the growth inhibition wad was estimated using the following equation:

$$\text{Percentage\, Inhibition}=\left[\frac{\text{Control}-\text{Treatment}}{\text{Control }}\right]\times 100$$

#### Antioxidant efficiency of ZnO–NPs

The Anti-oxidant activity of ZnO–NP was evaluated using the 1,1 diphenyl, 2 pyridyl-hydrazine (DPPH) assay in terms of free radical scavenging activity (RSA) [56]. The RSA of synthesized ZnO–NPs was tested by using the DPPH (1,1-diphenyl-2-pyridylhydrazine) assay. Until the DPPH test was required, 4 mg of 0.02 mM DPPH was mixed in 100 ml of Methanol and stored at 4 °C. A 2 ml stock solution of synthesized ZnO–NPs was combined with 1 ml of methanol and different test concentrations (25 to 150 μg/ml). Ascorbic acid was used as a positive control in this experiment, whereas Methanol and DMSO were used as negative controls. All combinations were determined at 517 nm using a UV–vis spectrophotometer (Shimadzu UV 2600, Japan) after being incubated in the dark for 30 min. The color changes from Purple to Yellow when one DPPH electron pairs with a hydrogen atom from a strong antioxidant.

$$\text{Percentage\, DPPH\, Scavanging}=\left[\left\{1-\frac{\text{ABs}.\text{ sample}}{\text{ABs}.\text{ control}}\right\}\right]\times 100$$

The doses of synthesized NPs needed to Scavenge 50% of the Radicals were assessed using the linear Regression Curve (IC_50_).

#### Anti-hemolytic efficiency of ZnO–NPs

The hemolytic experiment was carried out using freshly extracted RBCs to assess the biocompatibility of produced ZnO–NPs. In this experiment, 2 ml of newly isolated human Erythrocytes were centrifuged for 10 min at 12,000 rpm. The RBC suspension was created by combining 4.9 ml of Phosphate Buffer saline with 100 μl of Erythrocytes [27]. After adding six different doses (ranging from 25 to 150 µg/ml) of bioinspired biosynthesized ZnO-NPs with a 100 μl erythrocyte solution, the combination was incubated for 60 min at 35 °C. Additionally, 15 min of 12,000 rpm centrifugation were completed. An Eliza reader operating at 540 nm was used to measure the hemoglobin release after that the resulting product was then transferred to a 96-well plate. The positive controls were Triton X-100 and DMSO. The preceding method was employed to measure the % hemolysis:

$$\text{Percentage\, hemolysis }=\left[\frac{\text{Sample\, absorbance}-\text{Neg}.\text{ control\, absorbance }}{\text{Pos}.\text{ control\, absorbance}-\text{Neg}.\text{ control\, absorbance }}\right]\times 100$$

#### Brine shrimps efficiency of ZnO–NPs

The brine shrimp lethality experiment was performed to evaluate in vitro cytotoxicity of synthesized zinc oxide nanoparticles [27]. Eggs of *Artemia salina* were incubated for 48 h under continuous aeration in 1000 ml of sterile saltwater. Once the Larvae have hatched, gather 10 of them and put them in a glass vial with sterilized salt water (4.5 ml). The Nauplius was then mixed with 0.5 ml of synthesized ZnO–NPs in every vial, all at different doses (ranging from 25 to 150 µg/ml). Vincristine sulfate, mature nauplii, and seawater were used in the vials that were used as positive controls, whereas containing nauplii, seawater, and DMSO were used in the vials that were used as negative controls. All vial was incubated for 24 h at room temperature with light. After a 24-h treatment period, the number of viable brine shrimp nauplii in every container was tallied, and the percentage of deaths was computed [57].

$$\text{Percentage\, Hemolysis}=\left[\{1-\frac{\text{sample}}{\textrm{control}}\}\right]\times 100$$

#### Statistical analysis

The ZnO–NPs synthesized utilizing green methods were tested for cytotoxicity, biocompatibility, antibacterial activity, and DPPH radical scavenging activity. The data are shown as mean values ± standard deviations from three independent replicates per sample. Software like Origin Lab, GraphPad Prism, and Microsoft Excel were used for statistical analysis.

## Results

### Identification of cyanobacterial strain *Desertifilum* sp. TN-15 under light microscope observation

**Fig. 2:**
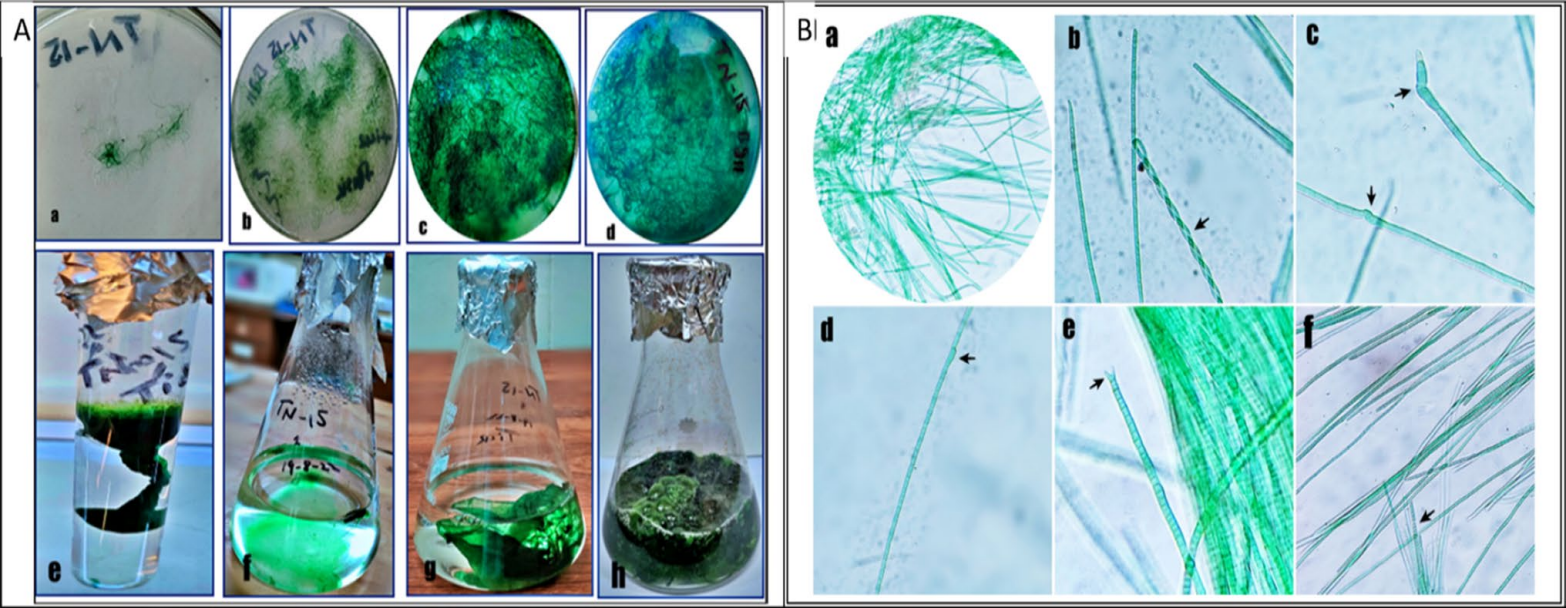
**A** Standard culturing phase of cyanobacterial strain *Desertifilum* sp. TN-15 (a – h) **a** In 3-days dark blue-green type initial growing filaments formed on the plate surface **b** After 10-days Green, pale and blue type filaments spreading on the plate surface **c** After 15-days Dark-green type dense filaments showed on the plate surface **d** After 20-days Filaments formation as a slime-dense aggregation type on the plate. **e** In 7-days growth of filaments in test tube (50 ml) as a clumps type formed **f** After10-days initial spreading growth of filaments in flask (500 ml) **g** After 20-days dense type growth of filaments formed in flask (500 ml) (h) After 30-days clumps type filaments growth in flask (500 ml) to formed biomass. **(B)** Morphological observation of Cyanobacterial strain *Desertifilum* sp. TN-15 under light microscope (a–f) **a** Entangled filaments showed **b** Twisted type and bent end of filaments **c** Colorless swollen hooked type end and swollen between the filaments **d** Between the filament cell arrangement differ as compare to other cells arrangement **e** Mature type extrusions at the end of filament **f** Rounded, conical-rounded, bent or tapered ends filaments and Filament break the sheath to form the trichomes with colorless sheath

Isolated cyanobacterial strain *Desertifilum* sp. TN-15 was purified and characterized morphologically [46], and also helpful in purification with the standard culturing method [58]. The isolated cyanobacterial Strain *Desertifilum* sp. TN-15 shows various diagnostic characteristics like; Colony (Table 1): Thallus dark green, pale green, or dark blue-green filamentous forming mats (colony). Filaments: Bule-green in color, non-heterocysts, solitary or entangled, straight, wavy, spiral or twisted types, and sometimes between and at the end of filaments swollen forming filaments observed. Trichomes: Cylindrical, or slightly constricted at cross-wall, with a thin sheath colorless, motility present. Cells: Mainly 2.6–4.4 μm wide and length of internal cross-wall cell; 4–5.2 μm. Cell contents are homogenous, small granules. Apical cells: Rounded, conical-rounded, slightly hooked, bent, or tapered types and sometimes with extrusions. Aerotopes were absent (Table 2, Fig. 2A, B).

**Table 1:** Standard culturing phase of cyanobacterial strain *Desertifilum* sp. TN-15

Colony growth	Response of *Desertifilum* SP. TN-15 on agar plates OF BG-_11_
3–DAYS (A)	Dark blue-green type initial growing filaments on the plate surface
10–DAYS (B)	Green, pale and blue type filaments spreading on the plate surface
15–DAYS (C)	Dark-green type dense filaments showed on the plate surface
20–DAYS (D)	Filaments formation as a slime-dense aggregation type on the plate
Biomass formation	Response of *Desertifilum* SP. TN-15 IN broth media of BG-_11_
7– Days (E)	Growth of filaments in test tube (50 ml) as a clump type
10–days (F)	Initial spreading growth of filaments in the flask (500 ml)
20–Days (G)	Dense-type growth of filaments in the flask (500 ml)
30– Days (H)	Clumps type filaments growth in the flask (500 ml) to form biomass
References	This study

**Table 2:** Morphological features of Cyanobacterial strain *Desertifilum* sp. TN-15

Characteristics	*Desertifilum* SP. TN-15
Sample collection site	Jhang district, Punjab, Pakistan
Habitat	Rice field water (In summer season)
Filament color	Blue-green color
Filament type	Straight or slightly wavey, solitary or entangled, Sometimes Spiral type, and Sometimes between and at the end of filament cells swollen
Filament toward the end	Attenuated at the ends and sometimes with extrusions
Filament constriction	Marginally constricted at cross-wall
Sheath	Thin, colorless attached at the trichomes
Aerotopes	Absent
Necridic cells	Absent
Motility	Present
Apical cells	Rounded, conical-rounded, slightly hooked, bent, or tapered ends
Cell shape	Cylindrical, mainly longer than wide
Cell content	Homogenous, and small granules
cell width	2.6–4.4 μm
Cell length	4–5.2 μm
Reference	This study (According to Cellamare et al. [59])

**Fig. 3:**
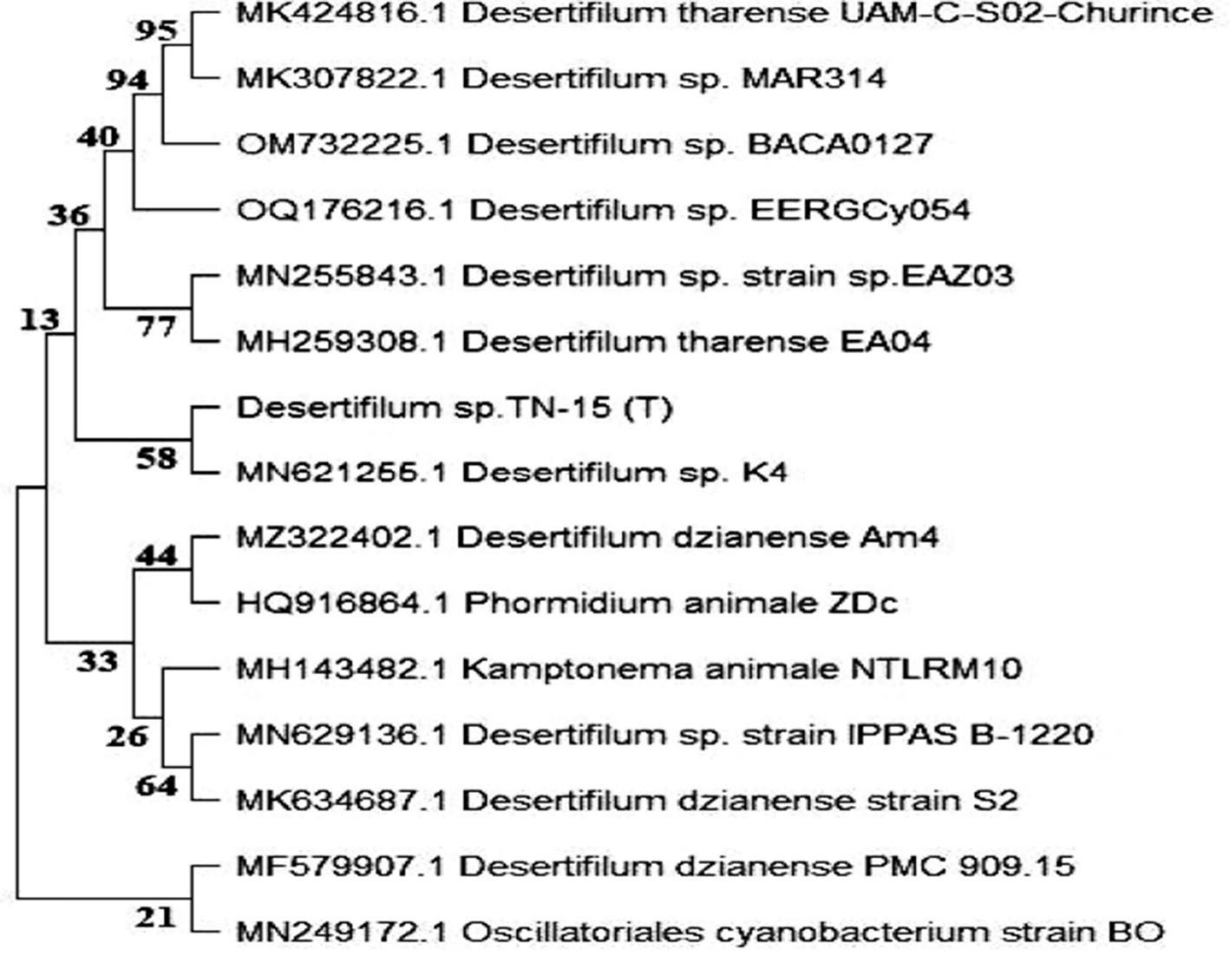
Evolutionary tree of cyanobacterial strain *Desertifilum* sp. TN-15 according to 16S rRNA gene sequencing

The cyanobacterial isolate strain *Desertifilum* sp. TN-15 was identified through molecular analysis of its 16S rRNA gene sequences to be a member of the *Desertifilum* genus. The solitary cyanobacterial strain is currently identified as *Desertifilum* sp. TN-15 (Fig. 3).

### Characterization of biosynthesized zinc oxide nanoparticles (ZnO–NPs) from cyanobacterial strain *Desertifilum* sp. TN-15

#### UV–vis spectrophotometer analysis

**Fig. 4:**
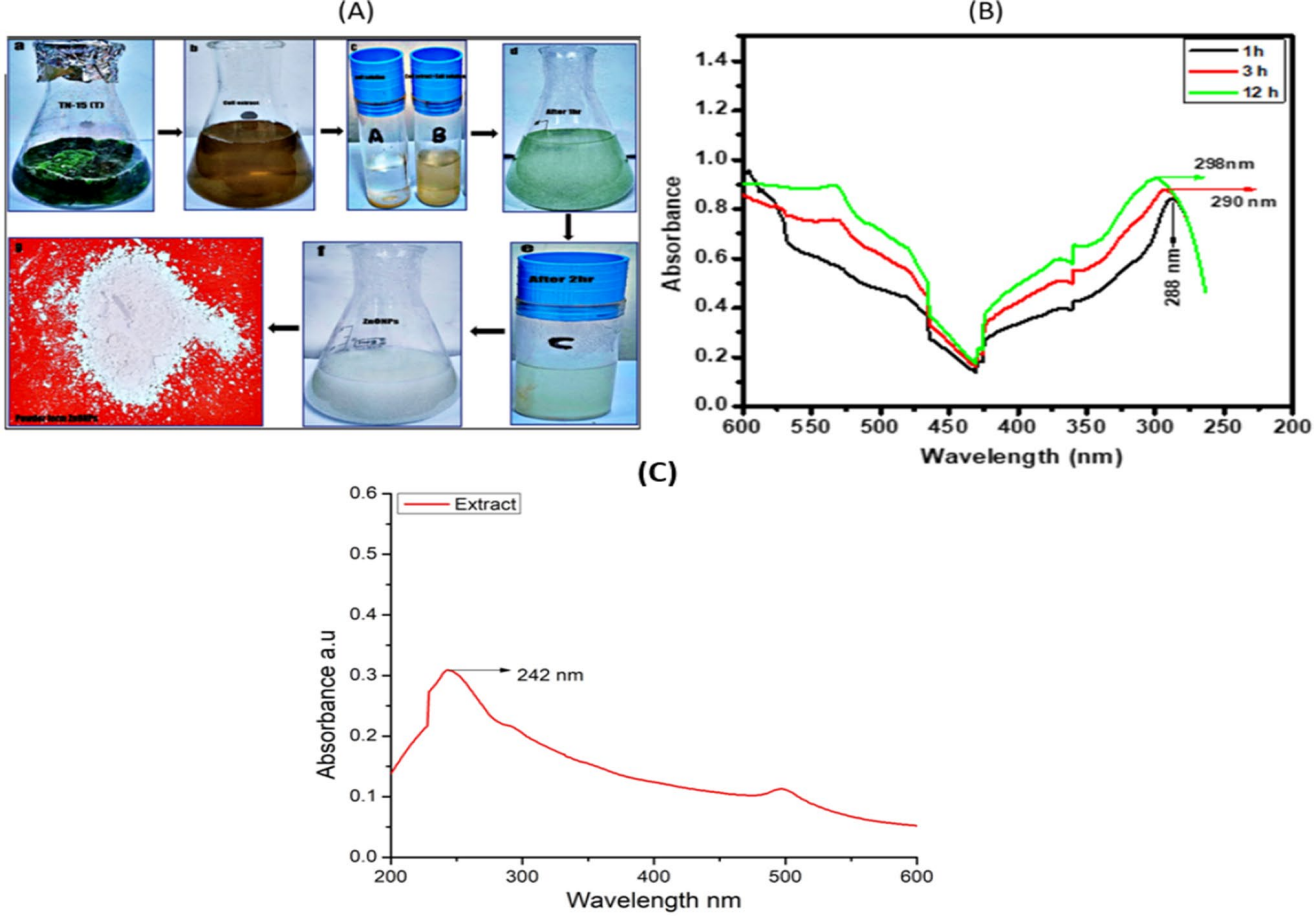
**A** Visual observation of biosynthesized ZnO–NPs from cyanobacterial strain *Desertifilum* sp. TN-15 (**a**–**g**) **a** Algal biomass of *Desertifilum* sp. TN-15 **b** Cell extract after filtration **c** **A**) Salt solution of Zinc acetate dehydrates **B**) Cell extract and Salt solution **d** After 1 h on hot plate it appears green color solution **e** After 2 h pale white color solution appears **f** After 12 h, ZnO–NPs formed pure white color formation in open air **g** The pellets dried, and formed ZnO–NPs in white color powder. **B** UV.vis spectroscopy of ZnO–NPs **C** UV.vis spectroscopy of *Desertifilum* sp. TN-15 cell extract

In this work, zinc oxide nanoparticles (NPs) were biosynthesized using cyanobacterial extract from *Desertifilum* sp. TN-15, which served as both reducing and stabilizing agents. Advances in ZnO–NPs development were made possible by a variety of characterization methods. By monitoring a color shift after the addition of zinc acetate to the cyanobacterial extract at 60 °C, the synthesis of biosynthesized zinc oxide nanoparticles was verified. Surface plasmon resonance was identified as the cause of the change from dark green to turbid white (Fig. 4A) [49]. A 40-min sonication of 1 mg/ml solutions was performed to evaluate the stability of the zinc oxide NPs. The turbid colloidal solution was left to stabilize for 12 h, during which time surface plasmon resonance was recorded at various intervals. The solution was examined at 200–800 nm in wavelengths. The stability of the colloidal solution at 12 h was shown by the UV spectra, which showed an absorption peak at 298 nm. The UV–vis spectrum is shown in Fig. 4(B,C).

#### Fourier transform infrared spectroscopy (FTIR)

Synthesized zinc oxide nanoparticles (ZnO–NPs) from the algal extract of the cyanobacterial strain *Desertifilum* sp. TN-15 were subjected to FTIR spectra analysis, which provides important new information about the different bioactive chemicals involved in the nanoparticle creation process. The functional groups that are present and their possible functions in the stabilization and reduction of ZnO–NPs are thoroughly understood according to the observed distinctive peaks. The bond stretching of O=C=O is responsible for the prominent peak at 2349 cm^−1^, which indicates the presence of carbonyl groups [50]. The presence of a strong connection is significant because it indicates that carbonyl compounds and ZnO–NPs interact significantly, which may help stabilize the nanoparticles in the aqueous solution. The wide peaks seen between 3700 and 3610 cm^−1^, more precisely between 3700 and 3610 cm^−1^, are indicative of stretching of the O–H bond [17]. These peaks correspond to alcohols, which may have a role in stabilizing and capping the nanoparticles. These peaks' medium sharpness indicates the presence of hydroxyl groups, which can strengthen the stability of the nanoparticles by forming hydrogen bonds with them. The peak, located at 1520 cm^−1^, is indicative of a nitro compound possessing a robust N–O stretching bond [32]. The ability of nitro compounds to withhold electrons is well-known, and this ability may help stabilize the nanoparticles by preventing agglomeration. The peak at 1080 cm^−1^ shows that primary alcohols have substantial C–O bond stretching [28]. The existence of these functional groups raises the possibility that primary alcohols contribute to ZnO–NP reduction by supplying the electrons required to convert Zn^+2^ ions into ZnO nanoparticles. Furthermore, the band at 895 cm^−1^ is indicative of a stretched alkene C–H bond [51]. The peak located within the 895–885 cm^−1^ range suggests the existence of alkenes, which could potentially interact with the surface of the nanoparticles and enhance their stability. Alkanes' C-H bending vibrations are generally represented by a peak at 665 cm^−1^ [51]. This peak suggests that the interaction between alkanes and ZnO–NPs may also involve alkanes. By supplying a protective organic layer, the absorption of these molecules on the surface of the nanoparticles can further improve ZnO–NP stability.

**Fig. 5:**
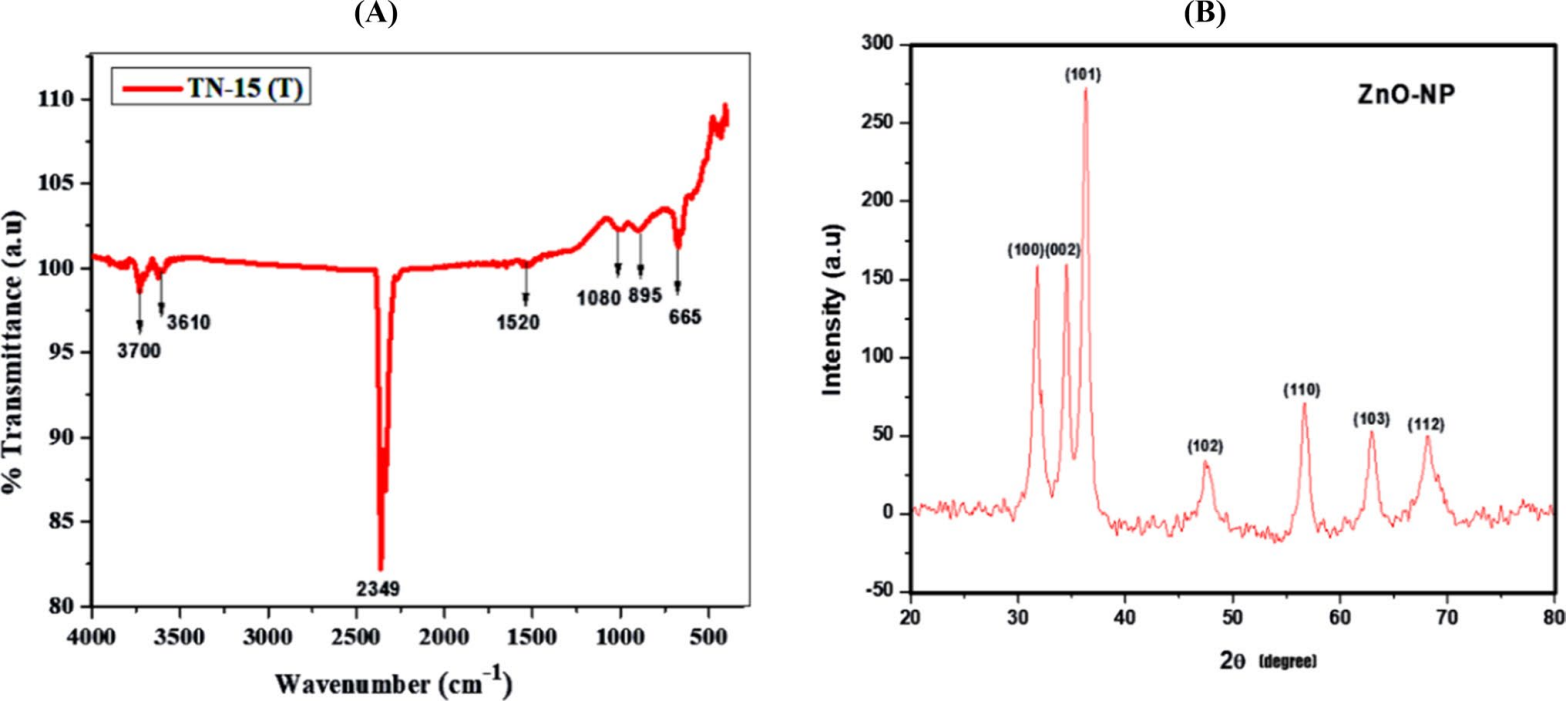
**(A)** FTIR of biosynthesized ZnO–NPs from cyanobacterial strain *Desertifilum* sp. TN-15 **(B)** X-Ray Diffraction (XRD) pattern of biosynthesized ZnO–NPs

The cyanobacterial strain *Desertifilum* sp. may have released protein molecules as a result of these functional groups' interaction with zinc oxide nanoparticles. ZnO–NP synthesis and stabilization are significantly influenced by TN-15. These naturally occurring compounds interact with the surface of the nanoparticles to stabilize and reduce them, which stops them from aggregating and improves their dispersibility in aqueous solutions (as displayed in Fig. 5(A).

#### X-ray diffraction (XRD) analysis

The produced zinc oxide nanoparticles (ZnO–NPs) can be thoroughly analyzed using X-ray diffraction (XRD) to learn more about their purity and crystalline structure [14]. Specifically, the crystal planes (100), (002), (101), (102), (110), (103), and (112) correspond to the diffraction peaks found at 2θ angles of 31.60°, 34.90°, 36.50°, 47.50°, 57.41°, 62.62°, and 67.65°. These peaks represent the hexagonal quartzite structure, which is a typical ZnO crystalline form (Fig. 5B). The ZnO–NPs' crystalline size was estimated to be 46 nm by applying the Debye-Scherer formula and taking into account the diffraction peaks' widening [52]. The successfully synthesized ZnO–NPs conform well with typical ZnO crystal structures, as confirmed by the reference pattern (JCPDS card number 89–7102). The XRD pattern's strong, distinct peaks show that the ZnO–NPs are extremely crystalline [53]. These peaks' clarity and intensity suggest that the crystal structures are well-defined and have few flaws. ZnO–NPs have a variety of uses, and this crystallinity is crucial because it frequently corresponds with advantageous qualities like optical and electrical capabilities. The produced ZnO–NPs appear to be quite pure, as indicated by the lack of any other notable peaks in the XRD pattern. It is assumed that the sample is free of major contaminants and secondary phases because there are no superfluous peaks. Applications where impurities could negatively impact the properties and performance of ZnO–NPs require this purity.

#### Zeta potential analysis

**Fig. 6:**
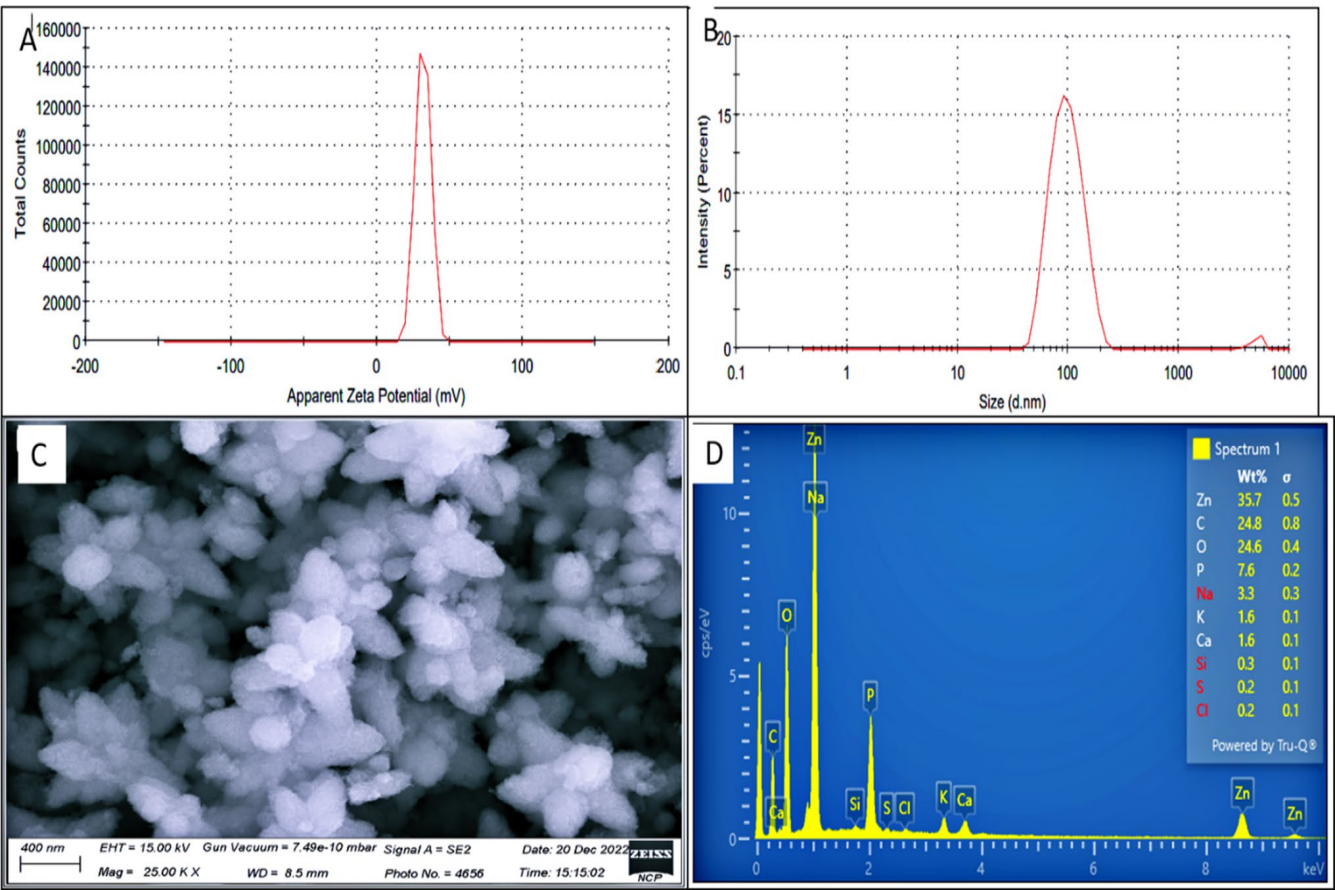
**A** Zeta potential distribution of biosynthesized ZnO–NPs indicates the positive surface charge **B** Size distribution and mean particle size of biosynthesized ZnO–NPs as 94.80 nm **C** SEM picture of biosynthesized ZnO–NPs. **D** Analysis of synthetic ZnO–NPs elemental composition as revealed by EDX

The zeta potential of the synthesized zinc oxide nanoparticle was + 31.6 mV, suggesting that it is more soluble in liquids, stable, and has a positive surface charge. The average particle size of the produced ZnO–NPs was 94.80 nm, with a particle size variation spanning from 70 to 100 nm (Fig. 6A, B) (Tables 3, 4).

**Table 3:** Conditions for Zeta Potential (ZP) Measurements

Conditions for ZP measurements	
Buffer name	Phosphate-buffered saline
Dispersant RI	1.330
pH	10.1
Viscosity (cP)	0.8872
Dispersant dielectric constant	78.5
Temperature (°C)	25.0
Zeta runs	12
Count rate (kcps)	39.8
Measurement position (mm)	4.50
Cell description	Zeta dip cell
Attenuator	6

**Table 4:** Zeta Potential Measurements of *Desertifilum* sp. TN-15 derived biogenic ZnO–NPs

Zeta size distribution, zeta potential and PDI	Results
Z-Average size (d.nm)	94.80
PDI	0.178
Intercept	0.966
Zeta potential (mV)	+ 31.6
Zeta deviation (mV)	5.19
Conductivity (mS/cm)	0.323
Result quality	Good

#### Scanning electron microscopy (SEM) and EDX analysis

The surface appearance and size of zinc oxide nanoparticles (ZnO–NPs), which were produced from the cyanobacterial extract of *Desertifilum* sp. TN-15, are shown in the SEM image (Fig. 6C). The nanoparticles, which have an average size of about 94.80 nm, have a characteristic star-shaped morphology. The cyanobacterial extract may have an impact on the direction of nanoparticle growth, as seen by this structure. Certain aggregation is observed, which is normal for nanoparticle synthesis even if the ZnO–NPs are comparatively equally dispersed. Because of their rough surface roughness, the star-shaped nanoparticles have a higher surface area and can be used in a variety of biological, sensing, and catalytic fields.

The chemical composition of synthesized ZnO–NPs was analyzed by energy-dispersive X-ray spectroscopy (EDX) analysis (Fig. 6D). The generated ZnO–NPs EDX investigation revealed several emission peaks for the elements Zn at 35.7%, C-24.8%, 0–24.6%, P-7.6%, Na-3.3%, K-1.6%, Ca -1.6%, Si-0.3%, S-0.2%, and Cl-0.2%. The biggest proportion of peaks, as well as the maximum emission of Zn, C, and O elements, show the compounds present in algal cell extract that are responsible for the creation of ZnO–NPs. These findings verified that zinc oxide nanoparticles had been successfully synthesized.

### Potential assessment of synthesized ZnO–NPs for biomedical applications

#### Antibacterial efficiency of ZnO–NPs

**Fig. 7:**
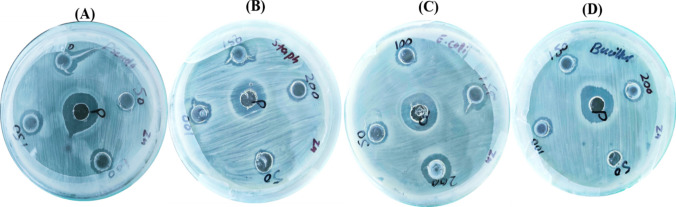
Antibacterial activity of biosynthesized Zinc nanoparticles determined by agar-well diffusion assay. Pictures shows inhibition zone produced by biosynthesized ZnO–NPs against different bacterial strains. The growth inhibition of important bacterial species at different concentrations of ZnO–NPs: **A*** P*. *aeruginosa* **B** *S*. *aureus* **C** E.coli **D** *B*. *subtilis*

**Fig. 8:**
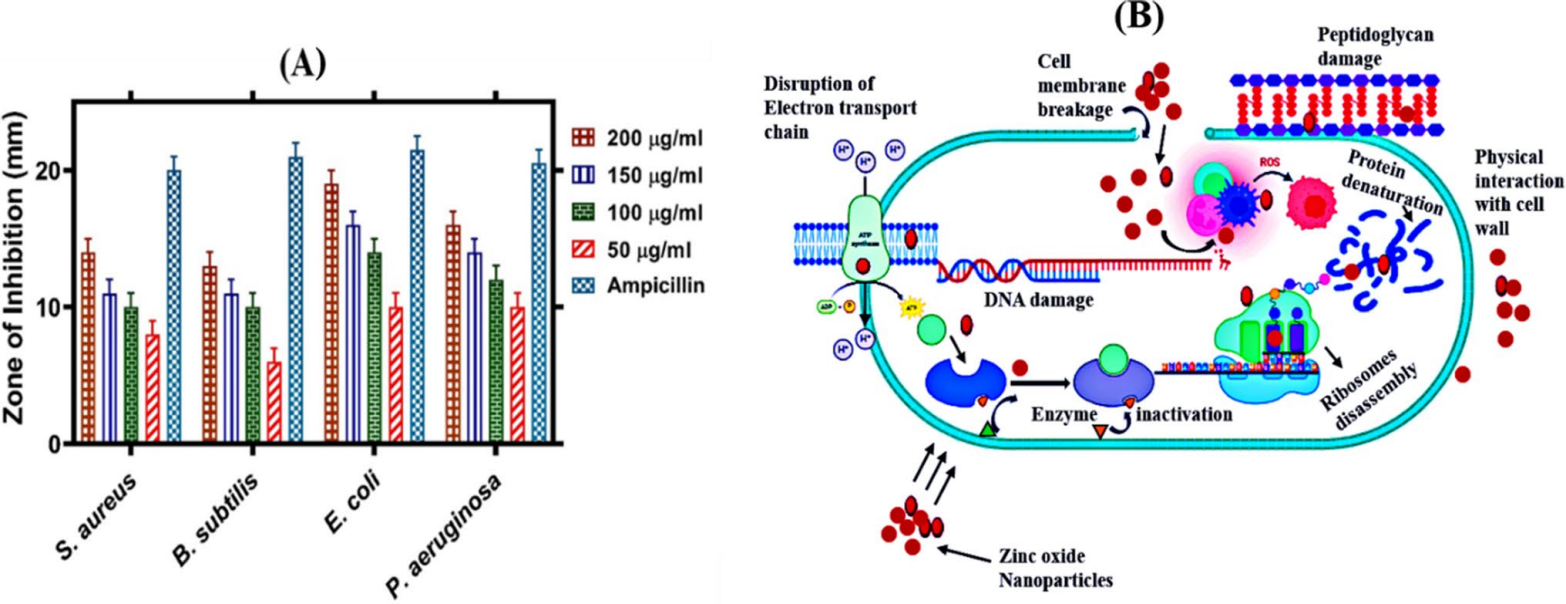
**A** Antibacterial potential of ZnO–NPs **B** Mechanism action of ZnO–NPs antimicrobial activity

The synthesized ZnO–NPs with *Desertifilum* sp. TN-15 were used against four different bacterial strains *Staphylococcus aureus, Escherichia coli, Bacillus subtilis,* and *Pseudomonas aeruginosa* on Luria–Bertani (LB) broth agar plate used as the positive control. This study used a concentration of 50–200 µg/ml of ZnO–NPs to produce good antibacterial activity against all tested bacteria. In this study ZnO–NPs with algal extract of *Desertifilum* sp. TN-15 has stronger antibacterial activity, as effective antibacterial activity was reached with only 200 µg/ml of the compound. Zinc oxide nanoparticles (ZnO–NPs) showed a considerable variety in their maximum % inhibition against different strains of bacteria during our investigation. The percentage inhibition varied between 94.7 ± 0.003 and 200 µg/ml for *Escherichia coli* and 70.8 ± 0.003 to 200 µg/ml for *Pseudomonas aeruginosa*. *Bacillus subtilis* and *Staphylococcus aureus* both displayed percentage inhibitions that ranged from 54.2 ± 0.003 to 200 µg/ml and 65.3 ± 0.003 to 200 µg/ml, respectively. Specifically, at 150 µg/ml, the inhibition percentages for *Pseudomonas aeruginosa*, *Bacillus subtilis*, *Escherichia coli*, and *Staphylococcus aureus* were 77.1 ± 0.003, 59.7 ± 0.003, 47.7 ± 0.003, and 43.1 ± 0.003, respectively; *Bacillus subtilis* and *Escherichia coli* were the least susceptible strains, with MIC values of 50 µg/ml. These results highlight the increasing antibacterial activity of ZnO–NPs at increasing concentrations, as illustrated in Figs. 7 and 8A and summarized in Table 5.

**Table 5:** MIC calculations of different Bacterial Strains and MIC Value of Fungal Strain

Bacterial strains	MIC (µg/ml)
Gram-positive	
*Staphylococcus aureus*	30.05 ± 0.003
*Bacillus subtilis*	15.3 ± 0.003
Gram-negative	
*Escherichia coli*	41.8 ± 0.003
*Pseudomonas aeruginosa*	37.5 ± 0.003
Fungal strain	MIC (µg/ml)
*Alternaria alternata*	15 ± 0.003

ZnO-NPs can generate reactive oxygen species (ROS) such as hydroxyl radicals, superoxide anions, and hydrogen peroxide when exposed to light or through intrinsic reactions. These ROS can cause oxidative stress in microbial cells, damaging cellular components like lipids, proteins, and DNA, ultimately leading to cell death. ZnO-NPs can interact with the cell membrane, causing physical disruption and increasing membrane permeability. This can lead to leakage of cellular contents and eventual cell lysis. The dissolution of ZnO-NPs can release Zn^2+^ ions, which can penetrate microbial cells and disrupt various intracellular processes. Zn^2+^ ions can interfere with enzyme function, protein synthesis, and genetic material, inhibiting microbial growth and reproduction (Fig. 8B).

#### Antifungal potential of ZnO–NPs

**Fig. 9:**
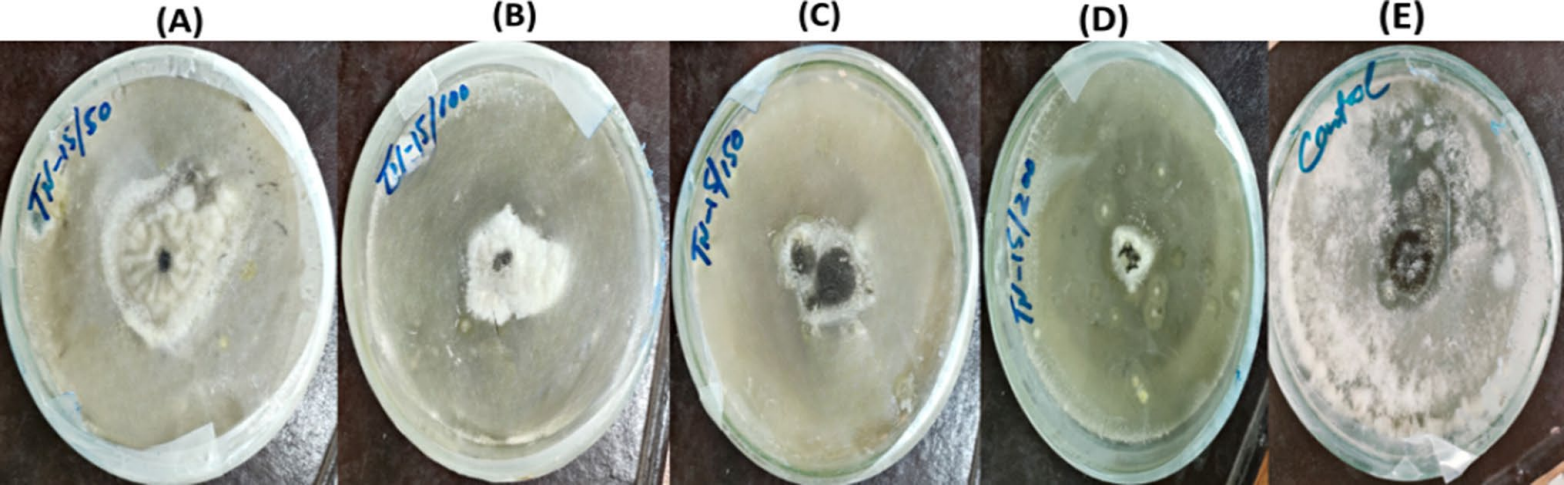
Antifungal Activity of ZnO–NPs determined by a food poisoning assay. Pictures shows inhibition zone produced by biosynthesized ZnO–NPs against *Alternaria alternata*. The growth inhibition of *Alternaria alternata* at different concentrations of ZnO–NPs **A** 50 µg/ml **B** 100 µg/ml **C** 150 µg/ml **D** 200 µg/ml and **E** Positive control

**Fig. 10:**
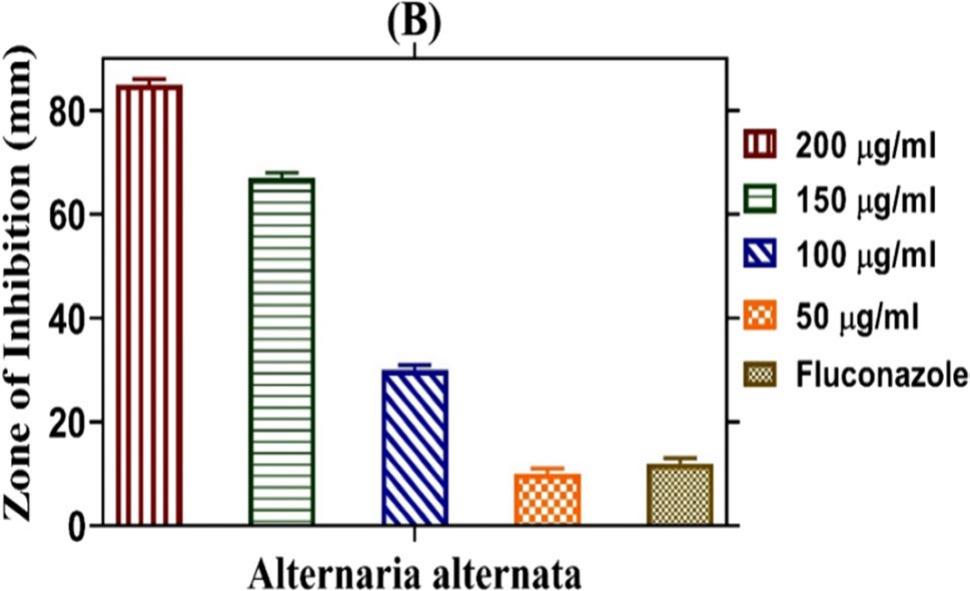
Antifungal potential of ZnO–NPs

In our study, biogenic zinc oxide nanoparticles (ZnO–NPs) were assessed for their antifungal capabilities against different fungal strains, with doses ranging from 50 to 200 µg/ml. The studied fungal strain showed % reduced development upon the presence of nanoparticles, as shown in Figs. 9 and 10. Mycelial growth was favorably inhibited by a greater dose of ZnO–NP, with 200 µg/ml of nanoparticles exhibiting the greatest growth suppression. There was a direct correlation between the concentration of zinc nanoparticles surrounding the well and their number. A value of 50 µg/ml was found to be the minimal inhibitory concentration (MIC). As indicated in Table 5, the maximum percentage inhibition against *Alternaria alternata* varied from 90 ± 0.5 at 200 µg/ml to 70 ± 0.03 at 150 µg/ml. According to our research, ZnO–NPs have the ability to effectively suppress fungal growth; the effects of the inhibitor are stronger at higher doses.

#### Antioxidant potential of ZnO–NPs

**Fig. 11:**
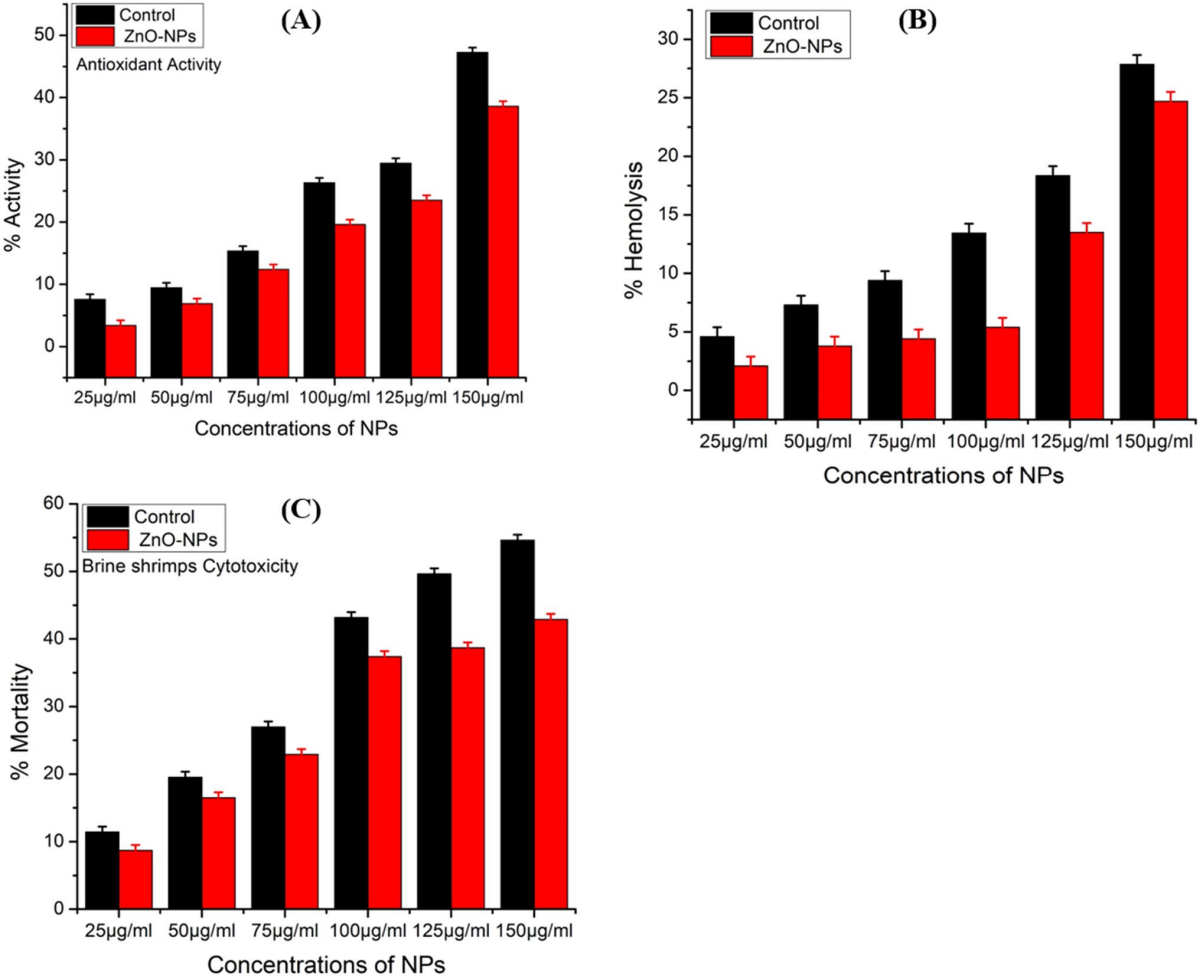
**A** Antioxidant potential of biosynthesized ZnO–NPs **B** Biocompatibility potential of biosynthesized ZnO–NPs against human RBCs **C** Cytotoxicity against *Artemia salina*

The ZnO–NPs effectiveness as an antioxidant is demonstrated in Fig. 11A. With different doses (ranging from 25 to 150 µg/ml), the antioxidant activity of ZnO–NPs and the nature of their antioxidant properties were assessed. At 150 µg/ml, the total antioxidant content reached its highest value of 38.61 ± 0.003. The findings showed that 25 µg/ml and 50 µg/ml doses showed the lowest IC_50_ of 3.40 ± 0.003 and 6.9 ± 0.003, respectively, indicating that they have less antioxidative potentials, while 75 µg/ml showed a considerable scavenging potential of 12.4 ± 0.003. Following, concentrations of 100 µg/ml at 19.6 ± 0.003, 125 µg/ml at 23.5 ± 0.003, and 150 µg/ml at 38.61 ± 0.003 showed good antioxidant capacity. The highest level of scavenging occurred at 150 µg/ml, and activity declined as concentration fell. Ascorbic acid served as the benchmark.

#### Antihemolytic potential of ZnO–NPs

Zinc oxide nanoparticles (ZnO–NPs) were tested against human RBC with blood group AB + to determine their biocompatibility and toxicological properties. The maximum hemolytic potential is shown in Fig. 11B. Biochemical compounds that exhibit minimal hemolysis (less than 2%) are classified as non-hemolytic, mild hemolysis (between 2 and 5%), and hemolysis (more than 5%) [60]. As a result, the hemolytic assay was employed to test the biocompatibility of ZnO–NPs against human RBCs at concentrations ranging from 25 to 150 µg/ml. A value of > 150 µg/ml was determined as the IC_50_. When the concentration was 100 µg/ml, hemolysis was detected at a rate of 5.4%, followed by 13.5% at 125 µg/ml, and 24.7% at 150 µg/ml, indicating a high hemolysis potential. Our research demonstrated that biosynthesized ZnO–NPs are non-hemolytic at low concentrations against RBCs, demonstrating their biocompatibility and non-hazardous nature.

#### Brine shrimps potential of ZnO–NPs

Brine shrimp lethality experiment was employed to examine the cytotoxic effectiveness of ZnO–NPs produced from *Desertifilum* sp. TN-15. Figure 11C shows the percentage mortality of ZnO–NPs at various concentrations. The ZnO–NPs were tested at six different doses (ranging from 25 to 150 µg/ml). The ZnO–NPs BSC experiment shows a dose-dependent effect. When compared to low concentration, high concentration is more deadly. Our findings showed that when concentration increases, mortality as a proportion rises as well.

## Discussion

The ability to synthesize nanoparticles using cyanobacteria (Blue-green algae) as a biomanufacturing plant is valuable since it allows for ecologically compatible production [61]. In addition to being affordable and energy-saving, it also creates innovative nanoparticles in various forms and sizes. Cyanobacterial nanoparticles can be used in a wide range of applications due to their diverse biological, physical, and chemical properties. Several investigations have demonstrated that these biosynthesized nanoparticles can operate effective photo-catalytic, antibacterial, and chemotherapy agents, among other purposes [61]. Cyanobacteria, often known as blue-green algae, need CO_2_ as a carbon source, light as an energy source, minerals, and water. In this investigation, an aqueous extract of blue-green algae (*Desertifilum* sp. TN-15 was used as a minimizing and stabilizing agent during the rapid and green biosynthesis of ZnO–NPs employing a reduced zinc acetate solution as a zinc precursor. According to Ebadi et al. [62], the white color of the metal nanoparticles is due to surface plasmon resonance phenomena. This would have confirmed the formation of ZnO–NPs [17].

Zinc oxide nanoparticles (ZnO–NPs) were synthesized biologically, and their quantitative production was observed by UV–visible absorption spectroscopy, which showed a clear absorbance peak at 298 nm. This finding is consistent with earlier research, including Elrefaey et al.'s, which indicates that the produced nanoparticles may have surface plasmon resonance (SPR). Surface plasmon resonance gives rise to the distinctive absorption peak seen in the UV–visible spectrum when free electrons on the surface of metal nanoparticles fluctuate together in reaction to input electromagnetic radiation. The existence of SPR indicates that ZnO–NPs were successfully synthesized and offers information about their optical characteristics, which are important for a number of uses including catalysis and sensing. Furthermore, ZnO–NP concentration in the solution may be quantitatively determined by measuring the strength of the absorption peak at 298 nm, which allows for accurate production oversight for particular uses [63]. Moreover, XRD examination demonstrated that ZnO–NPs are crystalline and star-shaped with a size of 46 nm. This outcome was consistent with earlier research in which the cyanobacterium *Nostoc* sp. EA03 was used to create ZnO–NPs [62]. Significant peaks in FTIR spectra of ZnO–NPs are attributable to vibratory stretching of the O–H, C–O, and C–H bonds. FTIR absorption peaks indicated the presence of C-H functional groups. The presence of the C–H group is indicated by the absorption peak at 895 cm^−1^. The signal at 3700 cm^−1^ confirms the presence of O–H groups. Thus, it is most likely that hydroxyl groups were added to alkanes to create and stabilize the nanoparticles. It is conceivable that heptadecane molecules could serve to be a model for a reaction that reduces zinc ions and generates ZnO–NPs. The capacity of functional groups in marine algal extracts to perform dual roles of bio-reduction and stabilization of ZnO–NPs was demonstrated through an IR spectroscopy analysis. Previous research [14, 17, 62] validated this finding, demonstrating the hydroxyl groups' capacity to interact with ZnO–NPs and provide a flexible tool for attaching diverse functions. Scanning electron microscopy (SEM) has offered further information on surface shape and the size of the nanoparticles [64]. In this work, ZnO–NPs were formed in star-shaped, small-width-to-length ratio nanoparticles. The SEM results were confirmed by Ebadi, et al. [62]. The energy-dispersive X-ray spectroscopy (EDX) method was utilized to verify the chemical makeup of the components. The three greatest percentage peaks that the synthesized ZnO–NPs released, representing zinc, carbon, and oxygen, validated the results. These results were confirmed by previous research on synthesized zinc oxide nanoparticles [37]. The synthesized zinc oxide nanoparticle had a positive surface charge, was stable, and was more soluble in liquids, as shown by its zeta potential of + 31.6 mV. The manufactured ZnO-NPs had a particle size distribution ranging from 70 to 100 nm, with a mean size of the particles of 94.80 nm. These expected sizes were in good accordance with the results of the zeta sizer and the Scherrer equation. As a result, our findings have the best stability (+ 31.6 mV). These results of zeta potential were confirmed by previous research on synthesized zinc oxide nanoparticles [37].

The findings demonstrated that ZnO–NPs, did not exhibit any discernible antibacterial action against the pathogens under study at low doses. ZnO–NPs can occasionally act as a source of sustenance for organisms in the environment. Zinc (Zn) is an essential trace element that serves as a cofactor for numerous vital biological macromolecules, including enzymes and structural proteins. This element is crucial for various cellular processes such as DNA synthesis, RNA transcription, cell division, and metabolism. As a result, microorganisms can utilize ZnO–NPs as a nutrient source to support their metabolic activities rather than experiencing inhibition of their growth.

This phenomenon can be explained by the ability of ZnO–NPs to release Zn^2 + ions, which microorganisms can uptake and incorporate into their cellular machinery. Consequently, at low doses, ZnO–NPs may promote microbial growth by providing an additional source of zinc, essential for their enzymatic functions and overall metabolic processes [65–67]. The method of diffusion on agar wells was utilized to evaluate the antibacterial effectiveness of synthesized ZnO–NPs against *Pseudomonas aeruginosa*, *Bacillus subtilis*, *Escherichia coli*, and *Staphylococcus aureus* at doses ranging from 50 to 200 μg/ml. Surprisingly, MIC testing findings showed that *Escherichia coli* was significantly more susceptible to the ZnO–NPs than *Bacillus subtilis*, *Pseudomonas aeruginosa*, and *Staphylococcus aureus*. These results indicate how different bacterial strains respond to ZnO–NPs, with Escherichia coli showing the greatest sensitivity of all the strains examined. Moreover, our results demonstrate ZnO–NPs' broad-spectrum antibacterial potential by showing a significant inhibitory activity against most bacterial species. Interestingly, this work supports earlier studies that reported ZnO–NPs' antibacterial properties using a variety of plant-based synthesis techniques, which adds to the validity of our findings. All of these findings point to ZnO–NPs' intriguing potential as potent antibacterial agents, which has ramifications for a range of environmental and biological applications [68, 69]. Zinc oxide nanoparticles (ZnO–NPs) have antibacterial and antifungal properties that are mediated by a complex network of interconnected mechanisms. Zinc oxide nanoparticles (ZnO–NPs) interact with bacterial cells to produce reactive oxygen species (ROS), which causes oxidative stress and antibacterial activity. ROS cause damage to biological structures, such as DNA and membranes, which kills bacteria cells. Zinc oxide nanoparticles (ZnO–NPs) have the ability to enter bacterial cells and damage internal structures while disrupting with essential biological functions like protein synthesis and metabolism. Furthermore, ZnO–NPs have a high surface area-to-volume ratio, which increases their contact with bacterial cells and maximizes their antibacterial effectiveness. Similar to this, ZnO–NPs' antifungal action results from their capacity to damage fungal cell membranes and obstruct biological functions. Zinc oxide nanoparticles (ZnO–NPs) cause membrane disruption when they come into contact with fungal cells, which allows internal components to leak out and ultimately results in cell death. Zinc oxide nanoparticles (ZnO–NPs) have the potential to enter fungal cells and have intracellular effects, which can seriously impair fungal viability by upsetting vital metabolic pathways. To enhance their antifungal properties, ZnO–NPs could potentially interact with chitin and β-glucans, which are components of fungal cell walls [70–72].

The biocompatibility and toxicity of ZnO–NPs were also assessed in comparison to human RBC. The findings of this study verify that ZnO–NPs are cytotoxic when they exceed 25 μg/ml but are not hemolytic when they are less than 10 μg/ml. The findings of ZnO–NPs mediated by *Desertifilum* sp. were discovered to be in line with previous studies of ZnO–NPs made with *Geranium wallichianum* [73]. Strong DPPH radical scavenging potentials of ZnO–NPs reported at 150 μg/ml. According to the findings, antioxidant compounds were implicated in the reduction and stabilization of ZnO–NPs utilizing the algal extract of *Desertifilum* sp. Our findings on ZnO–NPs are consistent with previous reports on plant and algae-mediated ZnO–NPs [63, 73]. The cytotoxicity analysis for brine shrimps was employed to verify the hemolytic ability of ZnO–NPs against recently hatched *Artemia salina*. The cytotoxicity label is assigned to our test specimen (ZnO–NPs) according to its IC_50_ values of 35.37 μg/ml. The findings obtained utilizing ZnO–NPs from *Desertifilum* sp. were verified by prior research employing ZnO–NPs from *Alstonia scholaris* stem bark and red algae of *H. musciformis* [74, 75].

## Conclusion

In conclusion, this study highlights the significant potential of a newly discovered cyanobacterial strain *Desertifilum* sp. TN-15 in the synthesis of zinc oxide nanoparticles (ZnO–NPs) and its multifaceted applications in various fields. Through a polyphasic approach, we successfully isolated and characterized *Desertifilum* sp. TN-15 from a rice field revealing its unique properties and previously untapped potential. The biogenically synthesized ZnO–NPs demonstrated remarkable characteristics, including a surface plasmon resonance peak at 298 nm, confirmed involvement of biomolecules in the synthesis and stability, star-shaped morphology with a size of 80 nm, and crystalline nature with a crystalline size of 46 nm. Additionally, the ZnO–NPs exhibited favorable physical stability, potent antibacterial and antifungal activities, significant antioxidant properties, and notable antihemolytic effects on red blood cells. Furthermore, cytotoxicity assessment revealed low toxicity with an IC_50_ value of 3.1 µg/ml in brine shrimps. Our findings suggest that *Desertifilum* sp. TN-15 represents a promising candidate for the environmentally friendly synthesis of ZnO–NPs with potential applications in pharmacological and therapeutic fields. This investigation addresses a significant research gap by being the first to investigate *Desertifilum* sp. TN-15.

## Data Availability

The data that support the findings of this study are available from the corresponding author upon request.
